# Clinical Outcomes Based on the Attainment of Low-Density Lipoprotein Cholesterol Targets in Patients with Acute Coronary Syndrome in Real-World Practice

**DOI:** 10.1155/2022/2292379

**Published:** 2022-12-17

**Authors:** Wei-Chieh Lee, Yi-Hsuan Tsai, Yun-Yu Hsieh, Yen-Nan Fang, Chih-Yuan Fang, Po-Jui Wu, Huang-Chung Chen, Ping-Yen Liu, Hsiu-Yu Fang

**Affiliations:** ^1^Institute of Clinical Medicine, College of Medicine, National Cheng Kung University, Tainan, Taiwan; ^2^Division of Cardiology, Department of Internal Medicine, Chi Mei Medical Center, Tainan, Taiwan; ^3^Division of Cardiology, Department of Internal Medicine, Kaohsiung Chang Gung Memorial Hospital, Chang Gung University College of Medicine, Kaohsiung, Taiwan; ^4^Biostatistics Center, Kaohsiung Chang Gung Memorial Hospital, Kaohsiung, Taiwan; ^5^Division of Cardiology, Department of Internal Medicine, National Cheng Kung University Hospital, College of Medicine, National Cheng Kung University, Tainan, Taiwan; ^6^Division of Cardiology, Department of Internal Medicine, Jen Ai Chang Gung Health International Medical Center, Taichung Branch, Taichung, Taiwan

## Abstract

**Objective:**

A target of low-density lipoprotein cholesterol (LDL-C) <70 mg/dL or ≥50% reduction should be set. This study aimed to explore the information required to attain the optimal goal of lipid control for patients with ACS in real-world practice using big database analysis.

**Methods:**

Patients with ACS were enrolled between January 2005 and December 2019, and their medical history was obtained from the Chang Gung Research database. According to the attainment of LDL-C levels, the study population was divided into groups with and without ≥50% reduced LDL-C levels. In the group that achieved ≥50% reduced LDL-C levels, the study population was subdivided into groups with and without achievement of LDL-C level < 70 mg/dL.

**Results:**

This study enrolled 14,520 participants, out of whom only 3,367 patients (23.2%) achieved ≥50% reduced LDL-C levels. At the 3-year follow-up periods, higher incidences of cardiovascular (CV) mortality and all-cause mortality were absorbed in patients without ≥50% reduced LDL-C levels, especially in subgroups of hypertension and diabetes mellitus (DM). When comparing different percentages of reduced LDL-C levels, the significantly lowest hazard ratio (HR) of CV and all-cause mortality was noted at ≥50% reduced LDL-C levels (CV mortality; HR: 0.64; all-cause mortality; HR: 0.57).

**Conclusion:**

In the ACS population, better clinical outcomes were yielded in patients with ≥50% reduced LDL-C levels, especially in the hypertension and DM populations. However, strict lipid control did not show better clinical outcomes in patients with ≥50% reduction and <70 mg/dL in LDL-C levels.

## 1. Background

Hyperlipidemia is a major risk factor for coronary artery disease (CAD), and it is well known that treatment of hyperlipidemia reduces the morbidity and mortality of CAD [1]. In both Asian and non-Asian populations, the risk of acute coronary syndrome (ACS) is associated with an increase in low-density lipoprotein cholesterol (LDL-C) [2]. Therefore, lowering LDL-C and achieving a target of optimal levels of LDL-C are important for both primary and secondary intervention settings [3]. Current European Society of Cardiology/European Atherosclerosis Society joint guidelines emphasize that LDL-C remains the most important marker to attain treatment targets; regardless of the patient's current LDL-C levels, a target of LDL-C < 70 mg/dL or ≥50% reduction (if the baseline is between 70 and 135 mg/dL) should be set to treat patients with ACS [4]. However, treatment targets are difficult to achieve even with moderate or high-intensity statin use in real-world practice, especially in patients with ST-segment elevation myocardial infarction (STEMI) and renal insufficiency [5–8]. However, clinical outcomes were similar, including postmyocardial infarction (MI) angina, target vessel revascularization, and recurrent MI, between LDL-C target achievers and nonachievers in patients with STEMI in real-world practice [6]. However, LDL-C level was highly correlated as an indicator of nutritional status, and malnutrition is common in CAD patients and strongly correlates with increased long-term mortality [9–11]. Low LDL-C levels may represent underlying malnutrition and are related to the paradox of nutritional status [12]. Diet patterns and sex also influenced lipid concentrations [13, 14].

Owing to the gap in lipid control between randomized control studies and real-world practice, this study aimed to explore the information required to attain an optimal goal of lipid control for patients with ACS in real-world practice using big database analysis.

## 2. Methods

### 2.1. Patient Population

Patients with ACS were enrolled in our study from January 2005 to December 2019, and their medical history (including detailed laboratory values and drug use) was obtained from the Chang Gung Research Database (CGRD), which is the largest healthcare system in Taiwan. Patients with age ≥ 18 years and diagnosed with ACS (International Classification of Diseases, ninth revision, Clinical Modification (ICD-9-CM) code 410.xx, 411.xx, and 412.xx, or tenth revision (ICD-10) codes I20, I21, and I22) were included in the study. Patients were divided into two groups, with and without ≥50% reduced LDL-C levels; patients with ≥50% reduced LDL-C levels were further subdivided into ≥ and <70 mg/dL LDL-C levels.

Data on general demographics, comorbidities, baseline and follow-up LDL-C levels, medication use, cardiovascular (CV) mortality, and all-cause mortality of patients were obtained and compared between the abovementioned groups at a 3-year follow-up period. All comorbidities, including hypertension, diabetes mellitus, peripheral arterial occlusive disease, chronic obstructive pulmonary disease, ESRD, liver cirrhosis, a prior history of gastrointestinal bleeding, and stroke, were based on the discharge ICD code and/or associated medical treatment.

### 2.2. Ethical Statement

This retrospective study conforms to the ethical guidelines of the 1975 Declaration of Helsinki. This study was approved for human research by the Institutional Review Committee of the Kaohsiung Chang Gung Memorial Hospital (approval number: 202101055B0).

### 2.3. Definition

CV mortality is defined as death from arrhythmia, MI, or heart failure. All-cause mortality is defined as death from any cause.

### 2.4. Study Endpoint

The attainment of the LDL-C treatment goal was defined as ≥50% reduced LDL-C level. The study endpoints were CV and all-cause mortality at the 1-year and 3-year follow-up periods.

### 2.5. Statistical Analyses

The data are presented as the mean ± standard deviation, or numbers (percentages). The continuous variables of clinical characteristics of the two groups were compared using the independent samples *t*-test for accepting normal distribution and Mann–Whitney *U* test for rejecting normal distribution. The categorical variables of the clinical characteristics of the two groups were compared using the chi-square test. The time risks for CV and all-cause mortality between groups were compared using a Cox proportional hazards regression model, and the risks in terms of hazard ratios (HRs), CV mortality, and all-cause mortality were compared at different decreasing percentages of LDL-C levels and at different LDL-C concentrations. Kaplan–Meier curve analysis was performed using the log-rank test for CV and all-cause mortality in the groups during the 3-year follow-up period. The level of statistical significance was set at *p* < 0.05. All analyses were performed using SAS version 9.4 (SAS Institute, Inc., Cary, NC, USA).

## 3. Results

The comparison of baseline characteristics and clinical outcomes between the patients with and without ≥50% reduced low-density lipoprotein cholesterol levels.

This study enrolled 14,520 participants. Their baseline characteristics and clinical outcomes are shown in Table 1. A total of 3,367 patients (23.2%) achieved the LDL-C treatment goal (with ≥50% reduction in LDL-C level), and 11,153 patients (76.8%) did not achieve the LDL-C treatment goal (without ≥50% reduction in LDL-C level). In the patients with ≥50% reduced LDL-C levels, a younger age, a higher prevalence of males, and a higher value of body mass index were noted when compared to the patients without ≥50% reduced LDL-C levels. A higher prevalence of smoking and diabetes mellitus was noted in patients with ≥50% reduced LDL-C levels. A lower prevalence of hypertension, ESRD, and prior stroke was noted in the patients with ≥50% reduced LDL-C levels. A higher prevalence of high-intensity statin (with vs. without; 81.29% vs. 52.94%; *p* < 0.001) and ezetimibe (with vs. without; 15.89% vs. 12.69%; *p* < 0.001) use was noted in the patients with ≥50% reduced LDL-C levels compared to those without ≥50% reduced LDL-C levels. In patients with ≥50% reduced LDL-C levels, higher baseline LDL-C level (with vs. without; 140.80 ± 39.26 mg/dL vs. 101.60 ± 36.20 mg/dL; *p* < 0.001), lower achieved LDL-C level (with vs. without; 55.61 ± 17.47 mg/dL vs. 85.78 ± 29.57 mg/dL; *p* < 0.001), and higher decreasing percentage (with vs. without; 60.22 ± 7.70% vs. 8.12 ± 47.05%; *p* < 0.001) were noted.

At 1- and 3-year follow-up periods, higher incidences of CV mortality (with vs. without; 1-year: 1.78% vs. 2.35%; *p*=0.050; 3-year: 4.31% vs. 5.54%; *p* < 0.001) and all-cause mortality (with vs. without; 1-year: 4.37% vs. 6.18%; *p*=0.050; 3-year: 11.91% vs. 16.42%; *p* < 0.001) were noted in the patients without ≥50% reduced LDL-C levels.

### 3.1. Kaplan–Meier Curve Analysis for All-Cause Mortality and Cardiovascular Mortality in Patients with and without ≥50% Reduced Low-Density Lipoprotein Cholesterol Levels during the 3-Year Follow-Up Period

**Figure 1:**
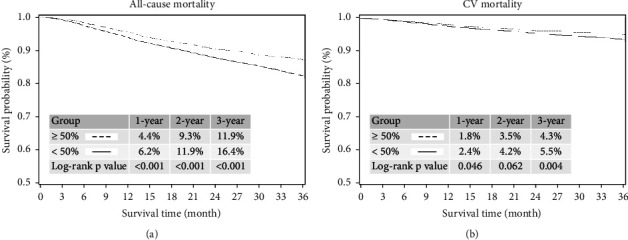
Kaplan–Meier curve analysis for all-cause and cardiovascular (CV) mortality in patients with and without ≥50% reduced LDL-C levels during the 3-yearfollow-up period. (a) During the 3-yearfollow-up period, a higher incidence of all-cause mortality was noted in patients without ≥50% reduced LDL-C levels (with vs. without; 1-year: 4.4% vs. 6.2%; *p* < 0.001; 2-year: 9.3% vs. 11.9%; *p* < 0.001; 3-year: 11.9% vs. 16.4%; *p* < 0.001). (b) During the 3-year follow-up period, a higher incidence of CV mortality was noted in patients without ≥50% reduction in LDL-C levels (with vs. without; 1-year: 1.8% vs. 2.4%; *p*=0.046; 2-year: 3.5% vs. 4.2%; *p*=0.062; 3-year: 4.3% vs. 5.5%; *p*=0.004).

During the 3-year follow-up period, higher incidences of all-cause mortality (with vs. without; 1-year: 4.4% vs. 6.2%; *p* < 0.001; 2-year: 9.3% vs. 11.9%; *p* < 0.001; 3-year: 11.9% vs. 16.4%; *p* < 0.001; (Figure 1(a))), and CV mortality (with vs. without; 1-year: 1.8% vs. 2.4%; *p*=0.046; 2-year: 3.5% vs. 4.2%; *p*=0.062; 3-year: 4.3% vs. 5.5%; *p*=0.004; (Figure 1(b))) were noted in patients without ≥50% reduced LDL-C levels.

### 3.2. Kaplan–Meier Curve Analysis for All-Cause Mortality and Cardiovascular Mortality in Patients with Hypertension, Diabetes Mellitus, End-Stage Renal Disease, and Prior Stroke between the Groups with and without ≥50% Reduced Low-Density Lipoprotein Cholesterol Levels during the 3-Year Follow-Up Period

**Figure 2:**
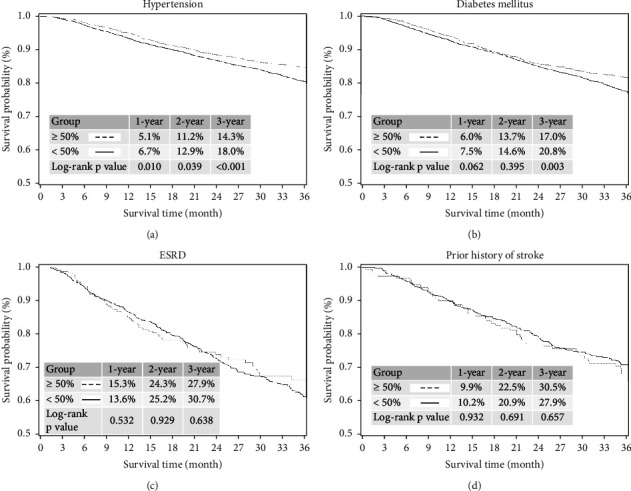
Kaplan–Meier curves analysis for all-cause mortality in patients with hypertension, diabetes mellitus (DM), end-stage renal disease (ESRD), and prior stroke between the groups with and without ≥50% reduced LDL-C levels during the 3-yearfollow-up period. (a) In the subgroup of hypertension, a higher incidence of all-cause mortality was noted in patients without ≥50% reduced LDL-C levels at the 3-yearfollow-up period (with vs. without; 1-year: 5.1% vs. 6.7%; *p*=0.010; 2-year: 11.2% vs. 12.9%; *p*=0.039; 3-year: 14.3% vs. 18.0%; *p* < 0.001). (b) In the subgroup of DM, a higher incidence of all-cause mortality was noted in patients without ≥50% reduced LDL-C levels at the 3-yearfollow-up period (with vs. without; 1-year: 6.0% vs. 7.5%; *p*=0.062; 2-year: 13.7% vs. 14.6%; *p*=0.395; 3-year: 17.0% vs. 20.8%; *p*=0.003). (c) In the subgroup of ESRD, the incidence of all-cause mortality did not differ between patients with and without ≥50% reduced LDL-C levels at the 3-yearfollow-up period (with vs. without; 1-year: 15.3% vs. 13.6%; *p*=0.532; 2-year: 24.3% vs. 25.2%; *p*=0.929; 3-year: 27.9% vs. 30.7%; *p*=0.638). (d) In the subgroup of patients with a prior stroke, the incidence of all-cause mortality did not differ between the patients with and without ≥50% reduced LDL-C levels at the 3-yearfollow-up period (with vs. without; 1-year: 9.9% vs. 10.2%; *p*=0.932; 2-year: 22.5% vs. 20.9%; *p*=0.691; 3-year: 30.5% vs. 27.9%; *p*=0.657).

In the subgroups of hypertension and diabetes mellitus (DM), a higher incidence of all-cause mortality was noted in patients without ≥50% reduced LDL-C levels at the 3-year follow-up period (Figures 2(a) and 2(b)). In subgroups of ESRD and prior stroke, a similar incidence of all-cause mortality was noted between the patients with and without ≥50% reduced LDL-C levels at the 3-year follow-up period (Figures 2(c) and 2(d)).

**Figure 3:**
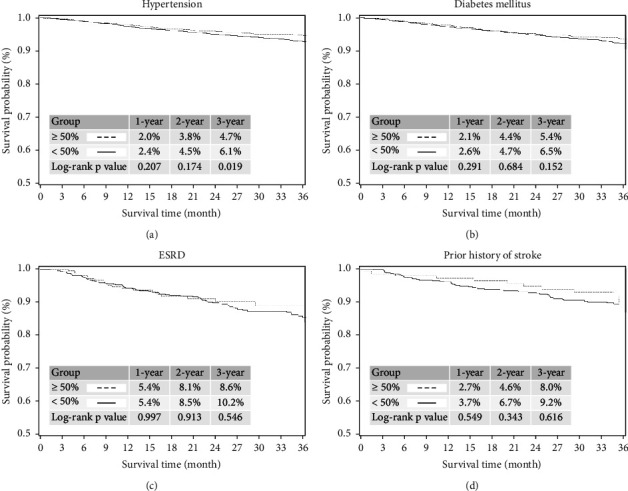
Kaplan–Meier curves analysis for CV mortality in patients with hypertension, DM, ESRD, and prior stroke between the patients with and without ≥50% reduced LDL-C levels during the 3-yearfollow-up period. (a) In the subgroup of hypertension, a lower incidence of CV mortality was noted in patients with ≥50% reduced LDL-C levels at the 3-yearfollow-up period (with vs. without; 1-year: 2.0% vs. 2.4%; *p*=0.207; 2-year: 3.8% vs. 4.5%; *p*=0.174; 3-year: 4.7% vs. 6.1%; *p*=0.019). (b) In the subgroup of DM, the incidence of CV mortality did not differ between the patients with and without ≥50% reduced LDL-C levels at the 3-yearfollow-up period (with vs. without; 1-year: 2.1% vs. 2.6%; *p*=0.291; 2-year: 4.4% vs. 4.7%; *p*=0.684; 3-year: 5.4% vs. 6.5%; *p*=0.152). (c) In the subgroup of ESRD, the incidence of CV mortality did not differ between the patients with and without a lowered LDL-C level (≥50%) at the 3-yearfollow-up period (with vs. without; 1-year: 5.4% vs. 5.4%; *p*=0.997; 2-year: 8.1% vs. 8.5%; *p*=0.913; 3-year: 8.6% vs. 10.2%; *p*=0.546). (d) In the subgroup of patients with a prior stroke, the incidence of CV mortality did not differ between the patients with and without ≥50% reduced LDL-C levels at the 3-yearfollow-up period (with vs. without; 1-year: 2.7% vs. 3.7%; *p*=0.549; 2-year: 4.6% vs. 6.7%; *p*=0.343; 3-year: 8.0% vs. 9.2%; *p*=0.616).

In the subgroup of hypertension, a higher incidence of CV mortality was noted in patients without ≥50% reduced LDL-C levels at the 3-year follow-up period (Figure 3(a)). In subgroups of DM, ESRD, and prior stroke, similar incidences of CV mortality were noted between the patients with and without ≥50% reduced LDL-C levels at the 3-year follow-up period (Figures 3(b)–3(d)).

### 3.3. Comparison of Baseline Characteristics and Clinical Outcomes between the Groups with and without Low-Density Lipoprotein Cholesterol Level <70 mg/dL and in Patients with ≥50% Reduced LDL-C Levels

Among patients with ≥50% reduced LDL-C levels, 2,741 (81.4%) patients achieved LDL-C levels of <70 mg/dL (Table 2). In the subgroup with ≥50% reduction and <70 mg/dL LDL-C levels, older age and a lower prevalence of smokers were noted; additionally, a higher prevalence of hypertension, DM, and ESRD was also noted. A similar prevalence of high-intensity statin use, and a lower prevalence of ezetimibe use were noted in the same subgroup. A lower baseline LDL-C level (with vs. without; 130.00 ± 31.27 mg/dL vs. 187.90 ± 35.78 mg/dL; *p* < 0.001), a lower achieved LDL-C level (with vs. without; 49.61 ± 12.18 mg/dL vs. 81.86 ± 12.06 mg/dL; *p* < 0.001), and a higher decreasing percentage (with vs. without; 61.21 ± 7.88% vs. 55.86 ± 4.82%; *p* < 0.001) were noted.

At the 3-year follow-up period, higher incidences of CV mortality (with vs. without; 4.74% vs. 2.40%; *p*=0.009) were presented in patients with ≥50% reduced and <70 mg/dL LDL-C levels. At 1-year and 3-year follow-up periods, higher incidences of all-cause mortality (with vs. without; 1-year: 4.78% vs. 2.56%; *p*=0.014; 3-year: 12.70% vs. 8.47%; *p*=0.003) were presented in the patients within the same subgroup.

### 3.4. Hazard Ratio of 3-Year Cardiovascular Mortality and All-Cause Mortality at Different Percentages of Reduced Low-Density Lipoprotein Cholesterol (LDL-C) Level and the Lowest LDL-C Level

When comparing different percentages of lowering LDL-C levels (Table 3), the significantly lowest HR of CV and all-cause mortality was noted at ≥50% reduced percentage of LDL-C (CV mortality; HR: 0.64; 95% CI: 0.53–0.78; *p* < 0.001; all-cause mortality; HR: 0.57; 95% CI: 0.51–0.64; *p* < 0.001). When comparing varyingly attained lowest LDL-C levels, a nonsignificant trend toward alower HR of CV and all-cause mortality was noted at the LDL-C level between 70 and 100 mg/dL (CV mortality; HR: 0.64; 95% CI: 0.35–1.20; *p*=0.164; all-cause mortality; HR: 0.84; 95% CI: 0.65–1.08; *p*=0.177).

## 4. Discussion

In real-world practice, the strict lipid target is hard to achieve. In one large cohort study, none of the patients at high CV risk achieved LDL-C<70 mg/dl for primary prevention and around 5% of patients at high CV risk achieved LDLC<70 mg/dl for secondary prevention. [15] The lipid paradox has been reported in patients with MI and malnutrition, and the benefits of strict lipid control may be limited in such a population [16, 17]. In our study, a total of 3,367 patients (23.2%) attained ≥50% reduced LDL-C levels and also developed lower CV and all-cause mortality when compared to those patients without ≥50% reduced LDL-C levels. Furthermore, only 2,741 patients (18.9%) could attain ≥50% reduction and <70 mg/dL in real-world practice. The patients with ≥50% reduced LDL-C levels had the lowest HR for all-cause mortality when compared to those with lowered LDL-C ratios of 30%–50%, 10%–30%, and <10%. Patients with the lowest LDL-C level (<70 mg/dL) did not have the lowest HR for all-cause mortality when compared to those with different LDL-C levels.

### 4.1. Lipid Paradox in Acute Coronary Syndrome

Several studies have reported a lipid paradox in patients with MI or ACS combined with cardiogenic shock. [18, 19] Lower LDL-C levels did not reflect better short-term outcomes owing to poor nutritional status and critical conditions. [12, 16] In the acute phase or critical condition of MI, large myocardial necrosis may cause heart failure, secondary liver failure, and give rise to the inflammatory phase, resulting in a drop in LDL-C that is observed immediately following an ACS event [20]. Therefore, a lower LDL-C level may not be suitable for all populations with ACS, especially those with critical conditions, severe heart failure, and malnutrition. [18] In our study, a decreasing percentage of LDL-C level (≥50%) provided a lower risk reduction at a 3-year follow-up period even if the patient presented with the lowest level of LDL-C pretreatment. Although strict lipid control is recommended for high-risk populations, real-world practice is variable and complex. Additionally, physicians must pay attention to the decreasing percentage of LDL-C levels, not the lowest value of LDL-C in real-world practice. However, a higher prevalence of comorbidities presented in patients with ≥50% reduction and LDL-C levels <70 mg/dL, which may have influenced the clinical outcomes.

### 4.2. Lipid Control for Patients with Concurrent Acute Coronary Syndrome, End-Stage Renal Disease, and Prior Stroke

In our study of subgroup analysis, ≥50% reduced LDL-C levels provided better clinical outcomes in patients with hypertension and DM, and strict lipid control could deliver long-term benefits in such a population. In patients with ESRD and a prior stroke, the incidence of all-cause mortality may not differ between groups with and without ≥50% reduced LDL-C levels. The relationship between LDL-C levels and stroke and the benefits of lipid control for patients with ESRD have been debated for many years. According to the recommendations of the guidelines for lipid control, it is reasonable to control the LDL-C target at <70 mg/dL to reduce the risk of major cardiovascular events in patients with ischemic stroke or transient ischemic attack, cerebral, or carotid atherosclerotic stenosis, or known CAD [21]. In patients with recent ACS and dyslipidemia, strict lipid control with proprotein convertase subtilisin kexin-9 inhibitor (PCSK9i) provides large absolute reductions in patients with polyvascular disease [22]. However, the cost-effectiveness of PCSK9i for such a population is hard to compare with other settings and needs to be evaluated in large longitudinal pharmacoeconomic studies [23]. Lipid paradox continues to exist in patients with acute ischemic stroke, and low acute-phase lipid levels are associated with high mortality [24, 25]. In patients with ESRD, some studies have suggested that high cholesterol is associated with lower mortality [26, 27]. In our study, the incidence of all-cause mortality did not differ between the groups with and without LDL-C levels of <70 mg/dL in patients with ≥50% reduced LDL-C levels.

### 4.3. Study Limitations

This study has several limitations. First, the study design was retrospective, and all information was obtained from the medical records. Second, the ICD-9-M and ICD-10-M codes only relied on the physician's choice in clinical practice and used medications. Third, a long study period was noted, and the target of the LDL-C threshold changed over the different periods. Fourth, the use of PCSK9i still had very strict limitations and these agents are not popularly used for patients without the attainment of the lipid-control goal in our healthcare system. Nevertheless, this study provides valuable information regarding lipid control in patients with ACS. Only of 23.2% patients could attain ≥50% reduced LDL-C levels, and only 18.9% could attain ≥50% reduction and <70 mg/dL LDL-C levels in real-world practice. This study also provides important information on lipid control for subgroups in real-world practice, including those with hypertension, DM, ESRD, and a prior stroke.

## 5. Conclusions

In the ACS population, better clinical outcomes were seen in patients with ≥50% reduced LDL-C levels, especially in the hypertension and DM populations. Strict lipid control did not show better clinical outcomes in patients with ≥50% reduction and <70 mg/dL LDL-C levels. The patients with ≥50% reduced LDL-C levels had the lowest HR for all-cause mortality when compared to those of patients with lowered LDL-C ratios of 30%–50%, 10%–30%, and <10%.

## Figures and Tables

**Table 1:** Baseline characteristics and clinical outcomes in the patients with and without decreasing LDL-C level ≥50%.

	Decreasing ≥ 50%	Decreasing < 50%	*p* value
Number	3367	11153	
General demographics			
Age (years)	61 (12.7)	65 (12.9)	<0.001
Male sex (%)	2611 (77.55)	8124 (72.84)	<0.001
BMI (kg/m^2^)	25.77 (3.89)	25.52 (4.22)	0.006
Comorbidities			
Smoking (%)	708 (21.03)	1947 (17.46)	<0.001
Hypertension (%)	1996 (59.28)	6891 (61.79)	0.009
Diabetes mellitus (%)	1492 (44.31)	4592 (41.17)	0.001
PAOD (%)	11 (0.33)	75 (0.67)	0.022
COPD (%)	88 (2.61)	455 (4.08)	<0.001
ESRD (%)	222 (6.59)	869 (7.79)	0.021
Liver cirrhosis (%)	22 (0.65)	138 (1.24)	0.004
Prior GI bleeding (%)	157 (4.66)	834 (7.48)	<0.001
Prior stroke (%)	151 (4.48)	598 (5.36)	0.044
Lipid lower agents (%)			
High-intensity statin (%)	2737 (81.29)	5904 (52.94)	<0.001
Ezetimibe (%)	535 (15.89)	1415 (12.69)	<0.001
LDL-C level (mg/dl)			
Baseline level (mg/dL)	140.80 ± 39.26	101.60 ± 36.20	<0.001
The lowest level (mg/dL)	55.61 ± 17.47	85.78 ± 29.57	<0.001
Decreasing percentage (%)	60.22 ± 7.70	8.12 ± 47.05	<0.001
Clinical outcomes			
1-year			
CV mortality (%)	60 (1.78)	262 (2.35)	0.050
All-cause mortality (%)	147 (4.37)	689 (6.18)	<0.001
3-year			
CV mortality (%)	145 (4.31)	618 (5.54)	0.005
All-cause mortality (%)	401 (11.91)	1831 (16.42)	<0.001
F/U period (years)	2.6 (0.7)	2.5 (0.8)	0.007

Data are expressed as the mean (standard deviation) or as a number (percentage). Abbreviation: LDL-C, low density lipoprotein-cholesterol; BMI, body mass index; PAOD, peripheral arterial occlusive disease; COPD, chronic obstructive pulmonary disease; ESRD, end stage renal disease; GI, gastrointestinal; CV, cardiovascular; F/U, follow-up.

**Table 2:** Baseline characteristics and clinical outcomes in the patients with decreasing LDL-C level ≥50% and with or without LDL-C level <70 mg/dL.

	<70 mg/dL	≥70 mg/dL	*p* value
Number	2741	626	
General demographics			
Age (years)	62 (12.8)	58 (11.9)	<0.001
Male sex (%)	2129 (77.67)	482 (77.00)	0.715
BMI (kg/m^2^)	25.78 (3.92)	25.74 (3.73)	0.845
Comorbidities			
Smoking (%)	550 (20.07)	158 (25.24)	0.004
Hypertension (%)	1670 (60.93)	326 (52.08)	<0.001
Diabetes mellitus (%)	1276 (46.55)	216 (34.50)	<0.001
COPD (%)	77 (2.81)	11 (1.76)	0.137
ESRD (%)	200 (7.30)	22 (3.51)	<0.001
Liver cirrhosis (%)	18 (0.66)	4 (0.64)	0.960
Prior GI bleeding (%)	134 (4.89)	23 (3.67)	0.193
Prior stroke (%)	132 (4.82)	19 (3.04)	0.052
Lipid lower agents (%)			
High-intensity statin (%)	2162 (78.88)	508 (81.15)	0.205
Ezetimibe (%)	286 (10.43)	86 (13.74)	0.017
LDL-C level (mg/dl)			
Baseline level (mg/dL)	130.00 ± 31.27	187.90 ± 35.78	<0.001
The lowest level (mg/dL)	49.61 ± 12.18	81.86 ± 12.06	<0.001
Decreasing percentage (%)	61.21 ± 7.88	55.86 ± 4.82	<0.001
Clinical outcomes			
1-year			
CV mortality (%)	53 (1.93)	7 (1.12)	0.164
All-cause mortality (%)	131 (4.78)	16 (2.56)	0.014
3-year			
CV mortality (%)	130 (4.74)	15 (2.40)	0.009
All-cause mortality (%)	348 (12.70)	53 (8.47)	0.003

The data are expressed as the mean (standard deviation) or as a number (percentage). Abbreviation: LDL-C, low density lipoprotein-cholesterol; BMI, body mass index; COPD, chronic obstructive pulmonary disease; ESRD, end stage renal disease; GI, gastrointestinal; CV, cardiovascular.

**Table 3:** Hazard ratio of 3-year CV mortality and all-cause mortality at different percentages of decreasing LDL-C level and the lowest LDL-C level.

Decreasing ratio	CV mortality			All-cause mortality		
	HR	95% CI	*p* value	HR	95% CI	*p* value
≥50%	0.64	0.53–0.78	<0.001	0.57	0.51–0.64	<0.001
50–30%	0.67	0.57–0.81	<0.001	0.59	0.53–0.66	<0.001
30–10%	0.82	0.67–1.00	0.045	0.79	0.71–0.88	<0.001
<10%	1	—	—	1	—	—

The lowest LDL-level (mg/dL)						
<50	1.00	0.52–1.90	0.990	1.22	0.94–1.59	0.140
50–70	0.77	0.41–1.44	0.414	0.87	0.68–1.13	0.293
70–100	0.64	0.35–1.20	0.164	0.84	0.65–1.08	0.177
100–130	0.70	0.36–1.35	0.285	0.91	0.70–1.19	0.496
130–150	1.11	0.53–2.35	0.783	0.95	0.69–1.31	0.760
≥150	1	—	—	1	—	—

Abbreviation: CV, cardiovascular; LDL-C, low density lipoprotein-cholesterol; HR, hazard ratio; CI, confidence interval.

## Data Availability

The data are available from the corresponding author upon reasonable request.
